# Optimization of the selection of suitable harvesting periods for medicinal plants: taking *Dendrobium officinale* as an example

**DOI:** 10.1186/s13007-024-01172-9

**Published:** 2024-03-16

**Authors:** Peiyuan Li, Tao shen, Li Li, Yuanzhong Wang

**Affiliations:** 1 College of Biology and Environmental Sciences of Hunan Province, Jishou University, Jishou, 416000 China; 2 Medicinal Plants Research Institute, Yunnan Academy of Agricultural Sciences, Kunming, 650200 China; 3 College of Chemistry, Biological and Environment, Yuxi Normal University, Yuxi, 653100 Yunnan China

**Keywords:** Medicinal plant, *Dendrobium officinale*, ATR-FTIR, ResNet, Harvesting period, Anticipate

## Abstract

**Background:**

*Dendrobium officinale* is a medicinal plant with high commercial value. The *Dendrobium officinale* market in Yunnan is affected by the standardization of medicinal material quality control and the increase in market demand, mainly due to the inappropriate harvest time, which puts it under increasing resource pressure. In this study, considering the high polysaccharide content of *Dendrobium* leaves and its contribution to today’s medical industry, (Fourier Transform Infrared Spectrometer) FTIR combined with chemometrics was used to combine the yields of both stem and leaf parts of *Dendrobium officinale* to identify the different harvesting periods and to predict the dry matter content for the selection of the optimal harvesting period.

**Results:**

The Three-dimensional correlation spectroscopy (3DCOS) images of *Dendrobium* stems to build a (Split-Attention Networks) ResNet model can identify different harvesting periods 100%, which is 90% faster than (Support Vector Machine) SVM, and provides a scientific basis for modeling a large number of samples. The (Partial Least Squares Regression) PLSR model based on MSC preprocessing can predict the dry matter content of *Dendrobium* stems with Factor = 7, RMSE = 0.47, R^2^ = 0.99, RPD = 8.79; the PLSR model based on SG preprocessing can predict the dry matter content of *Dendrobium* leaves with Factor = 9, RMSE = 0.2, R^2^ = 0.99, RPD = 9.55.

**Conclusions:**

These results show that the ResNet model possesses a fast and accurate recognition ability, and at the same time can provide a scientific basis for the processing of a large number of sample data; the PLSR model with MSC and SG preprocessing can predict the dry matter content of *Dendrobium* stems and leaves, respectively; The suitable harvesting period for *D. officinale* is from November to April of the following year, with the best harvesting period being December. During this period, it is necessary to ensure sufficient water supply between 7:00 and 10:00 every day and to provide a certain degree of light blocking between 14:00 and 17:00.

**Supplementary Information:**

The online version contains supplementary material available at 10.1186/s13007-024-01172-9.

## Background

There are about 750–900 genera of orchids (Orchidaceae), and their origin can be traced back to about 120 million years ago [1]. *Dendrobium* is one of the largest genera in the Orchidaceae family, and most of its species have important medicinal, economic, and ecological values, playing an important role in the health and wellness of people around the world [2]. *Dendrobium officinale* is the most researched and popular medicinal plant in the genus *Dendrobium*, with high commercial value and rich in chemical components and pharmacological activities, and is regarded as “the first of the nine immortal herbs” [3]. Among them, polysaccharide compounds are important active ingredients affecting the quality of *D. officinale*, accounting for 20–40% of the total compounds, with good antioxidant and anti-inflammatory effects [4]. Modern pharmacological studies have shown that *D. officinale* contains pectin with distinctive structural features, which is an important compound for protecting the human liver and a key factor in determining the chewing texture of *D. officinale* [5]. Notably, related studies have shown that *D. officinale* leaves have higher polysaccharide content than the stems, and there are records of folk minorities using them for prevention, treatment of diseases and body maintenance [6]. This has caused researchers to use both the stem and leaf parts of *D. officinale* as an important basis for measuring its quality and yield.

In China, Yunnan is known as the “Kingdom of Plants” with complex terrain and significant climate changes at different times [7]. The suitable harvesting period for *Dendrobium* is from November to April of the following year, and the dry matter content (DMC), yield, and accumulation of effective chemical components change with different growth times. Usually, in the harvesting of *Dendrobium*, the period with higher yield can only be selected based on individual subjective factors, resulting in missing the optimal harvesting period, this damages production and economic income [8]. Morphological data can be used to comprehensively assess the variation of *Dendrobium* production in different months, which can solve the problem of production assessment to a certain extent.

DNA barcoding, high-performance liquid chromatography, and powder microscopic identification are common methods for the identification of *Dendrobium* herbs and original plants [9]. The identification mainly contains the origin, species and harvesting period of *D. officinale*. The above methods rely on the experience of researchers, and chemical analysis is reagent-consuming, expensive, and has the potential risk of environmental pollution. Spectroscopy has the advantages of being non-destructive, rapid and efficient, and has gradually become an important research method for quality control and qualitative analysis of traditional Chinese medicine in recent years [10]. Fourier Transform Infrared Spectrometer (FTIR) has been reported to be more widely used, but it has problems with low apparent resolution and overlapping of the characteristic peaks [11]. Three-dimensional correlation spectroscopy (3DCOS) can transform complex spectral data into a more intuitive image form and is a technique to characterize spectral feature information by improving the apparent resolution to solve the problem of overlapping spectral bands. At present, combining ATR-FTIR with chemometrics can further accomplish the information recognition of different chemical types, and the common recognition models mainly include Partial Least Squares Discriminant Analysis (PLS-DA) and Support Vector Machines (SVM), among which SVM has a simple structure and strong generalization ability, and has a unique advantage in dealing with small amount of samples [12]. Deep learning plays an important role in the field of image recognition and is the main method currently used in the development of artificial intelligence research [13]. Convolutional Neural Networks (CNN), which include convolutional operations and deep structure, is a representative algorithm of deep learning [14]. Residual Neural Network (ResNet) formed by its improvement has unique advantages in target recognition and image classification. Scholars at home and abroad have achieved good experimental results by using this algorithm combined with 3DCOS to classify and recognize samples, indicating that this method has good potential for application in the field of classification and identification of species, origin and harvesting period. Recently, ATR-FTIR spectroscopy combined with multivariate analysis has been used to determine chemical content for quality control of medicinal plants, with the PLSR model being the most common predictive model [15]. DMC is a direct factor affecting yield and is positively correlated with polysaccharide content [16]. In addition, little research has been reported on the appropriate harvesting period for *D. officinale*.

To summarize, combining stems and leaves to evaluate the yield and at the same time, establishing a scientific and effective method to identify the optimal harvesting period is of great significance for *D. officinale* herb production and reducing economic losses. In this study, the first attempt was made to identify the harvesting period of *D. officinale* by ResNet modeling and combined with morphological data to provide a fast and effective method for yield control of *D. officinale* in different months. In addition, the DMC of *D. officinale* in different months was predicted by ATR-FTIR. The results of the study can provide new methods and ideas for future research on the optimal harvesting period of *D. officinale* and related medicinal and food plants, and can also avoid economic losses caused by improper selection of harvesting period.

## Methods

### Material collection and sample processing

**Fig. 1:**
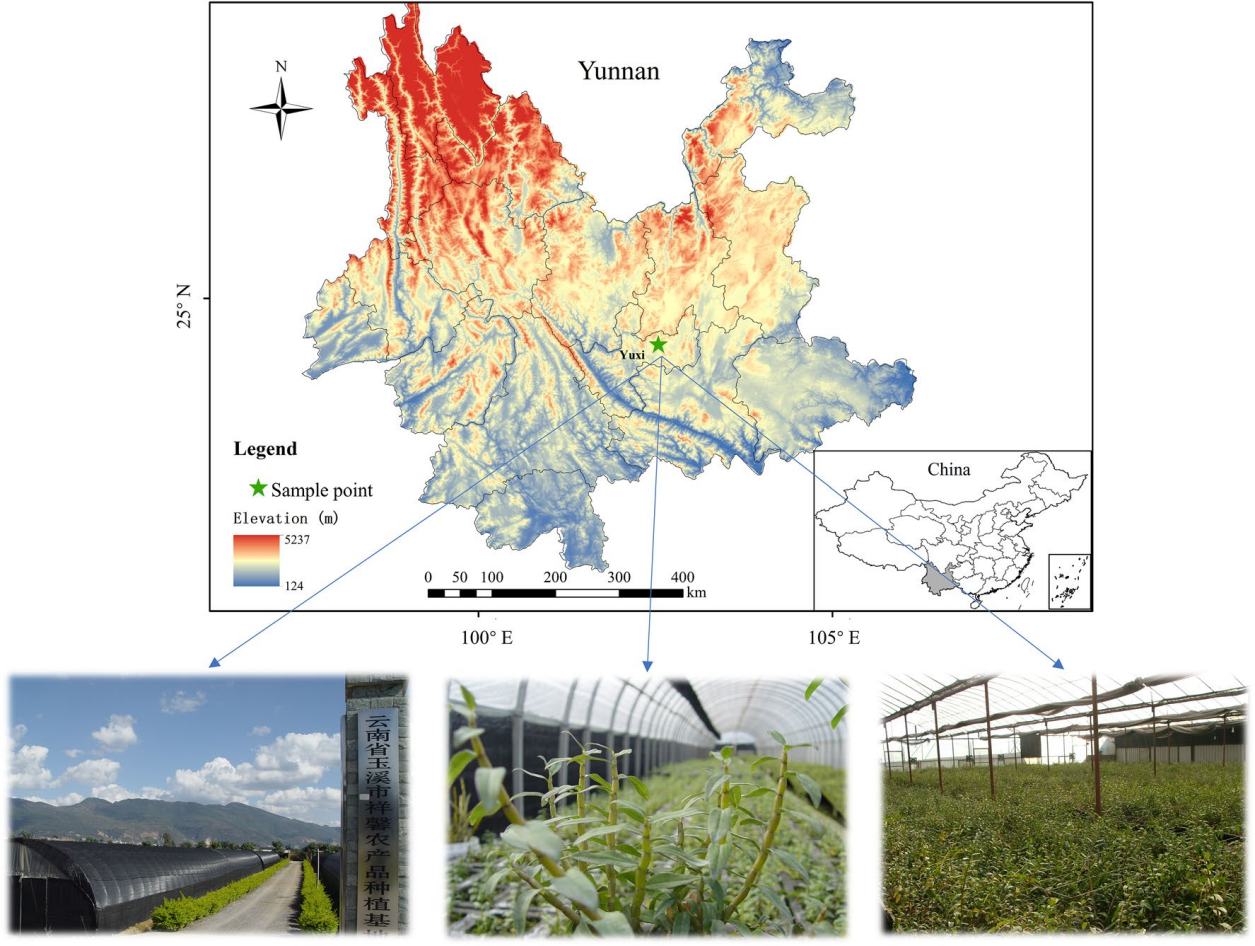
*D. officinale* planting base location

**Fig. 2:**
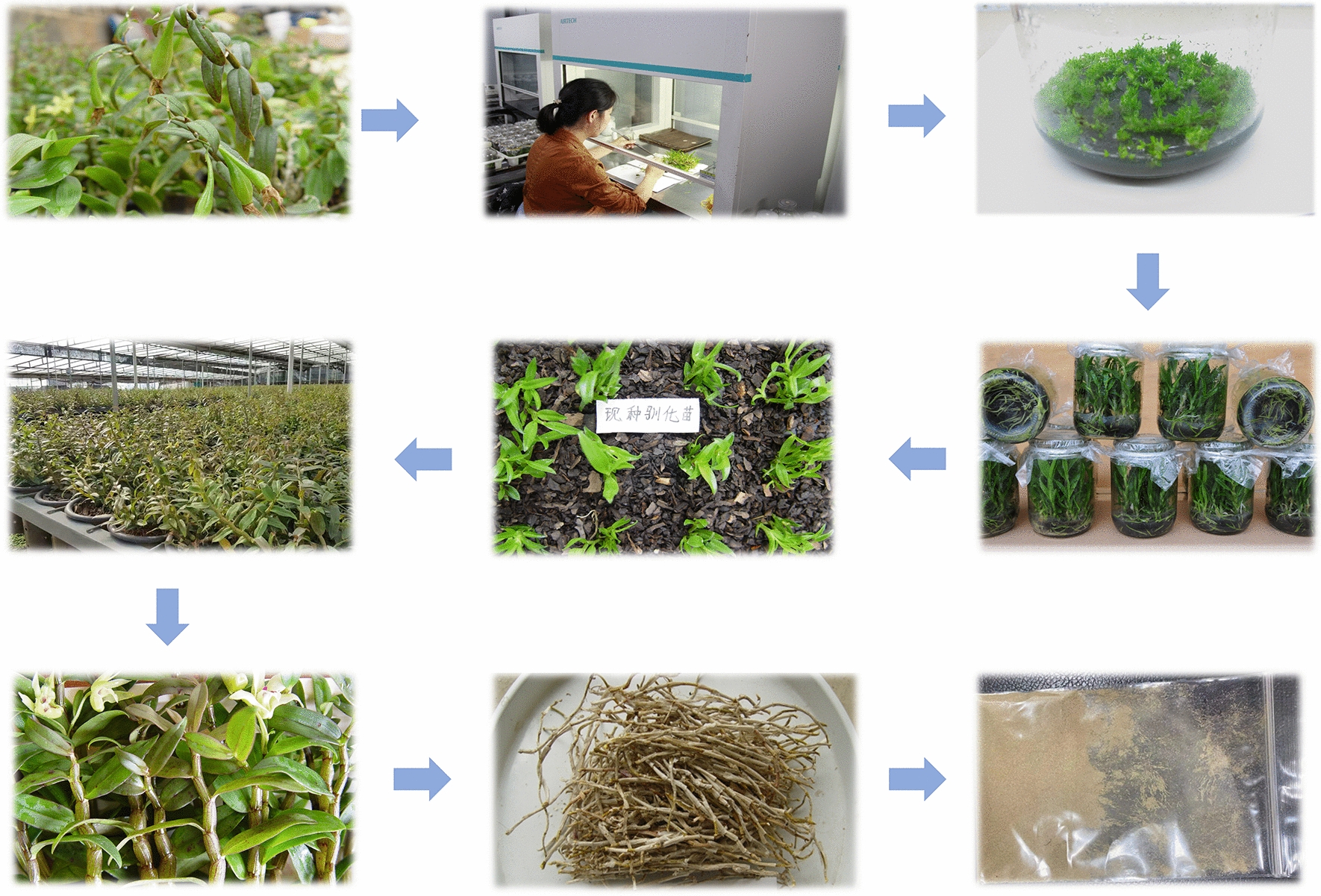
Sample Collection and Processing Procedures

The samples of *D. officinale* were collected from the lotus pond planting base in Beicheng Town, Hongta District, Yuxi City, Yunnan Province, and samples were collected at 15:30 on the 15th day of each month during the months of 1–12, with 12 individual plants sampled in each month and identified by Prof. Huang Hengyu of Yunnan University of Traditional Chinese Medicine (Fig. 1). Samples were cleaned after harvesting, divided into stem and leaf parts, length of the stem (X1, cm); fresh weight of stem (X2, g); fresh weight of leaf (X3, g); stem weights (X4, g); leaf weights (X5, g); dry matter content of stem (X6, %); dry matter content of Leaf (X7, %); water content of stem (X8, %) and water content of leaf (X9, %) were measured and calculated for subsequent analysis (Additional file 1: Table S1). Finally, the samples were dried to constant weight at 55 °C using an electric thermostat dryer (Shanghai Yiheng Scientific Instruments Co., Ltd.). The dried samples were ground using a portable high-speed grinder and passed through a 100-hole sieve, and the final sample powder was stored in a self-sealing bag for chemical analysis (Fig. 2).

### ATR-FTIR acquisition

Laboratory temperature and relative humidity were kept relatively constant, and sample powder spectral data were collected using a Fourier transform mid-infrared spectrometer with a deuterated triamcinolone sulfate crystal (DTGS) detector equipped with a single-reflector diamond universal ATR sampling accessory (UATR). In order to exclude the interference of H_2_O and CO_2_, spectral information of the background was collected prior to the acquisition of the sample spectra. The spectral range was 4000–450 cm^−1^ with a resolution of 4 cm^−1^ and 64 consecutive scans. Three replicate scans were performed for each sample, and the final data used for analysis were the average spectra of the three scans.

### Data preprocessing

FT-MIR spectrometer introduces redundant information and noise in addition to the feature information required for sample acquisition, which affects the results of subsequent analysis. Therefore, it is necessary to preprocess the raw spectral information before statistical analysis and modeling, and First Derivative (FD) and Second Derivative (SD) can overcome the overlap of spectral information and improve the resolution of overlapping peaks [17]. Multiple Scattering Correction (MSC) can solve the problem of absorbance shift by constructing a linear regression model [18]. Standard Normal Variable (SNV) can mitigate the ability to add or multiply in spectra [19]. In this study, the dataset was divided into training set (70%) and test set (30%) by the Kennard-Stone (KS) algorithm, and four methods, SD, MSC, SNV and SG, were selected for preprocessing. In addition, the above methods were performed by Matlab R2017a and SIMCA 14.1 software.

### 3DCOS acquisition

The theoretical foundations of the synchronous, asynchronous, and integrated 3DCOS generation methods are shown in Eqs. (1) to (5), where t denotes the perturbation interval, m denotes the number of spectral measurements, and the dynamic spectral intensity representation of the variable v is represented by the vectors [20].

1 $$S(v)=\left\{\begin{array}{c}s (v, {t}_{1})\\ s (v, {t}_{2})\\ \dots \\ s(v, {t}_{m})\end{array}\right.$$

The synchronous and asynchronous 3DCOS correlation strengths between v1 and v2 are denoted as Φ (*v1*, *v2*) and Ψ (*v1*, *v2*) (Eq. 2 Eq. 3) [21]. Respectively the integrated 3DCOS is obtained by multiplying the same synchronous and asynchronous 3DCOS*.*

2 $$\Phi \left({v}_{1},{v}_{2}\right)=\frac{1}{{\text{m}}-1}{N\left({v}_{1}\right)}^{{\text{T}}}\cdot N\left({v}_{2}\right)$$

3 $$\Psi \left({v}_{1},{v}_{2}\right)=\frac{1}{{\text{m}}-1}{Y\left({v}_{1}\right)}^{{\text{T}}}\cdot M\cdot Y({v}_{2})$$

4 $${\text{I}}\left({v}_{1},{v}_{2}\right)=\Phi \left({v}_{1},{v}_{2}\right)\cdot\Psi \left({v}_{1},{v}_{2}\right)=\frac{1}{{(m-1)}^{2}}\left[N{({v}_{1})}^{T}.N({v}_{2})\right]\cdot \left[N{({v}_{1})}^{T}\cdot M\cdot N({v}_{2})\right]$$

where N is defined as the Hilbert matrix Eq. (5).

5 $$N_{jk} = \left\{ {\begin{array}{*{20}c} {0,\quad j = k} \\ {\frac{1}{\pi k - j},\quad j \ne k} \\ \end{array} } \right.$$

### PLS-DA construction

PLS-DA is a supervised discriminative classification model in which the spectral data is X and the vector containing the category information is Y. The screening of variables contributing to the identification is based on the maximum covariance of X and Y. In this study, we have used PLS-DA as a model for the classification of samples. In this study, samples are assigned to categories (0 or 1) based on the predicted value of the dummy Y variable, and a Y value of 1 means that these samples belong to the category; a Y value of 0 means that these samples do not belong to the category [22].

Identification models for different harvesting periods (January-December) were developed based on ATR-FTIR spectra of both stem and leaf parts of *Dendrobium* officinale. Root mean square error (RMSEE), root mean square error of prediction (RMSEP) and root mean square error of cross-validation (RMSECV) were used as the evaluation indexes of the model accuracy; the closer the error value was to 0, the more stable the model was; R^2^ was used as a parameter to measure the match between the data and the model, and the closer the value was to 1, the more stable the model was; Q^2^ indicated the prediction ability of the model on new data, and in general, the model proved to have a good prediction performance when the value of Q^2^ > 0.5 proves that the model has good prediction performance. In addition, SIMCA 14.1 software was used to perform 200 substitution tests on the model to verify whether PLS-DA had overfitting problems.

### SVM model construction

SVM is a supervised classification model with good generalization ability, and its nonlinear algorithm can address the statistical validation deficiencies of PLS-DA in dealing with multiple covariates and inhomogeneous distributions, thus validating the results of PLS-DA [8]. Based on limited sample information, SVM has a unique advantage in solving high-dimensional patterns and nonlinear identification when the sample size is small [12]. A penalty coefficient (coast, *c*) that is too large or too small will result in poorer model generalization and risk of fitting; accompanied by an increase in the kernel function (gamma, *g*) and an increase in the number of support vectors, resulting in an impact on the training and prediction speed. The SVM model in this study was constructed by Matlab R2017b.

### ResNet model construction

ResNet can solve the problems of over-model weight decay, overfitting and gradient vanishing or gradient explosion caused by deepening of CNN layers [23]. Proposed by Microsoft Research in 2015. Compared to ordinary machine learning algorithms, ResNet avoids the errors of feature data extraction by artificial intelligence by using machines to automatically extract features to build models.

In this study, Conv block and Identity block were used to construct 14-layer ResNet to distinguish *Dendrobium* from different harvesting periods. Conv block was used when the size of the output F(x) is the same as the size of the input x and vice versa, Identity block was used. 60% of the training set was used to build the model and the minimum loss value was obtained by updating the weight values in conjunction with Stochastic Gradient Descent (SGD) to determine the convergence of the model. The stability and accuracy of the built model were verified using a 30% test set and finally 10% external validation set was fed to the built model to verify the generalization ability of the model.

### Statistical analysis

The coefficient of variation was calculated from the measured trait indicators, and factor analysis and combined factor scores were performed using online SPSSAU data analysis software (https://spssau.com/). Comparison of the weight share of each trait, the total factor scores were used to determine the appropriate harvesting period, and the DMC and coefficient of variation were used to determine the optimal harvesting period for yield. The coefficient of variation (1) and DMC (2) were calculated as follows:

6 $$\mathrm{CV }= \frac{x}{y}$$

7 $$\mathrm{DMC }= \frac{m}{n}\times 100\mathrm{\%}$$

In Eq. (1) CV is the coefficient of variation of each indicator, *x* represents the standard deviation of the indicator, and *y* represents the mean value of the indicator; in Eq. (2) DMC is the dry matter content of the samples in each month, *m* represents the dry weight, and *n* represents the fresh weight.

### Environment variable extraction

By importing the latitude and longitude of the sampling points in Yuxi City, Yunnan Province, into ArcGIS 10.0 software, and utilizing the Toolbox toolkit value extraction to point function, the values of solar radiation (sard) and average precipitation (Pre) were extracted and recorded from November to April of the following year (Table 1). Correlation (Spearman) analyses were conducted between the harvesting period of the samples (overwintering period, November–April of the following year) and the data (X1-X9) of different traits of *D. officinale*, comparing the effects of heat factor and moisture factor on its growth.

**Table 1:** Environmental and trait data for *Dendrobium* during the appropriate harvesting period

Month	Sard	Pre	X1	X2	X3	X4	X5	X6	X7	X8	X9
1 M	12780	13	18.26	5.86	3.55	1.08	0.56	18.53	15.7	81.47	84.3
2 M	15601	14	10.79	3.08	1.9	0.57	0.26	18.7	13.97	81.3	86.03
3 M	17831	48	23.49	4.11	3.41	0.83	0.46	20.53	14.04	79.47	85.96
4 M	19063	448	21.82	2.93	2.66	0.64	0.39	22.37	14.93	77.63	85.07
11 M	12533	11	32.69	6.98	6.16	0.99	0.76	14.27	12.5	85.73	87.5
12 M	11527	887	31.95	6.77	4.17	1.35	0.73	19.67	17.33	80.33	82.67

X1: Length of the stem (cm); X2: Fresh weight of stem (g); X3: Fresh weight of leaf (g); X4: Stem weights (g); X5: Leaf weights (g); X6: Dry matter content of stem (%);X7: Dry matter content of Leaf (%); X8: Water content of stem (%); X9: Water content of leaf (%); Sard: Solar radiation (KJ m^−2^ day^−1^); Pre: Precipitation (mm)

### Construction of predictive model

Partial Least Squares Regression (PLSR) modeling can correlate the changes in the spectral absorption intensity of a sample with its quantitative data, which can effectively quantify the quantitative data in the data, and a linear mathematical relationship between X (spectra) and Y (quantitative data) can be found by correlating the two sets of observed data [24]. In this study, the model performance of PLSR was evaluated by both linearity and accuracy; the calibration set samples were used to create and evaluate the model, and the remaining samples were the external validation set, where the model was considered to have a high degree of linearity when R^2^ was close to one. In addition, the residual prediction deviation (RPD) was used to further evaluate the model performance, where RPD < 1.4 indicated that the subspectral data were difficult to evaluate quantitatively, and 1.4 < RPD < 2.0 indicated that its spectral data could be evaluated quantitatively but the prediction accuracy needed to be improved [25]. RPD > 2.0 indicated that the model was effective and had a high prediction accuracy, and could be used for practical prediction. DMC is a direct factor affecting the yield of *D. officinale*, in this study, Matlab R2017a software was used to divide the dataset, and the PLSR models of *D. officinale* stems and leaves were established by The Unscrambler X 10.4 software, respectively, and the optimal model was compared to select the optimal model after predicting its DMC.

## Results

### Information on ATR-FTIR spectra of *D. officinale* stems and leaves

**Fig. 3:**
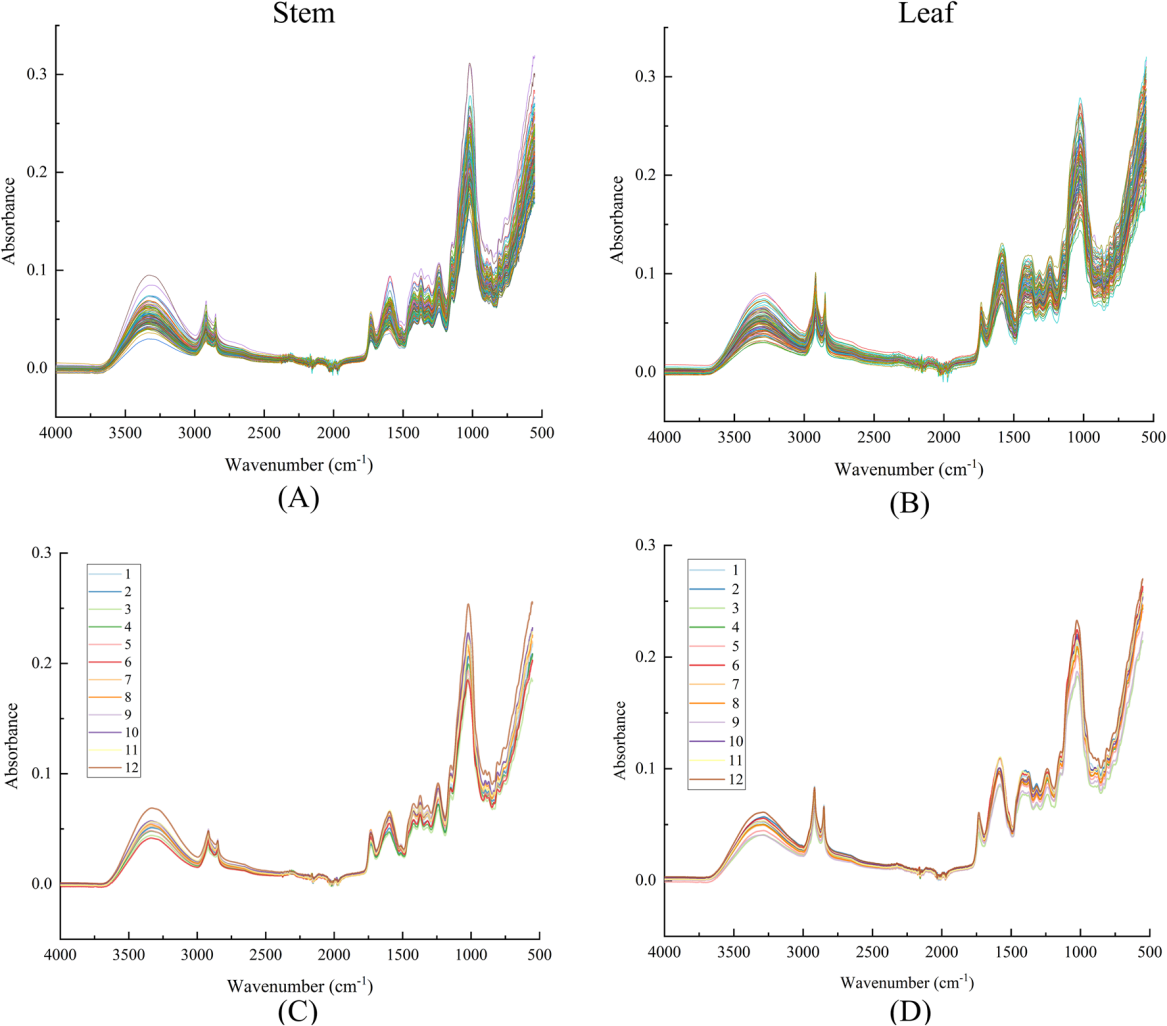
ATR-FTIR spectroscopy of *D. officinale* **A** and **B** Raw spectrum; **C** and **D** Average spectrum

The ATR-FTIR spectra of 120 stem and 120 leaf samples involved in the study were shown in Fig. 3. The spectral intensities of November-March were generally stronger than those of the other months, which might be caused by the fact that they were in the harvest period and the samples were relatively high in chemical content, with the strongest absorbance in December. The overall variation in stem and leaf spectra was small, with differences mainly in the range 3000–2750 cm^−1^ and near the spectral band 1595 cm^−1^.

### Classification results of PLS-DA

In the PLS-DA model, it could be seen that not all model results by preprocessing were improved, and all preprocessing methods make the model worse, except after SD, which is better (Table 2). Among them, the leaf (SD-L) was more accurate than the stem, with 100% and 97.22% accuracy in the training and test sets, respectively, and lower R^2^ and Q^2^, 0.7892 and 0.5611, respectively, and the PLS-DA model of the *D. officinale* stem with SD preprocessing (SD-S) was more robust compared to it, and there was no risk of overfitting (Additional file 1: Fig. S1). In addition, this preprocessing method was chosen for further modeling and analysis because the training and test sets of the PLS-DA model of SD-S were 97.62% and 97.22%, respectively, which still had the risk of misclassification.

**Table 2:** Parameters of the PLS-DA model

Style	LVs	R^2^	Q^2^	RMSEE	RMSECV	RMSEP	Accuracy of training test (%)	Accuracy of testing test (%)
Raw-S	10	0.511	0.313	0.203	0.229	0.198	86.9	77.78
**SD-S**	**15**	**0.875**	**0.616**	**0.104**	**0.190**	**0.133**	**97.62**	**97.22**
MSC-S	15	0.211	0.120	0.254	0.258	0.239	54.76	44.44
SNV-S	15	0.212	0.119	0.254	0.259	0.254	54.76	41.67
SG-S	9	0.415	0.236	0.243	0.241	0.231	78.57	61.11
Raw-L	11	0.536	0.307	0.121	0.213	0.178	86.9	77.78
SD-L	12	0.789	0.561	0.109	0.189	0.150	100	97.22
MSC-L	14	0.188	0.120	0.252	0.255	0.257	41.67	50
SNV-L	14	0.188	0.120	0.252	0.225	0.257	40.48	52.78
SG-L	10	0.449	0.228	0.186	0.226	0.211	84.52	72.22

Bolded values represent the models with the best results

### Discriminant results of SVM

The results of the SVM establishment of *D. officinale* stems by genetic algorithm (GA) were shown in Additional file 1: Fig. S2, based on the original data, the SVM model was established with a *c* = 98.902, *g* = 9.5 × 10^–4^, a training set accuracy of 57.14%, and a test set accuracy of 86.11%, which took 50.85 min; after SD preprocessing, the SVM model was established with a c value of 0.846, a g value of 10.0145, the training set accuracy was 80.95%, the test set accuracy was 88.89% took 117.26 min; due to the low accuracy, ResNet model is further selected for further analysis.

### The 3DCOS Information of *D. officinale*

**Fig. 4:**
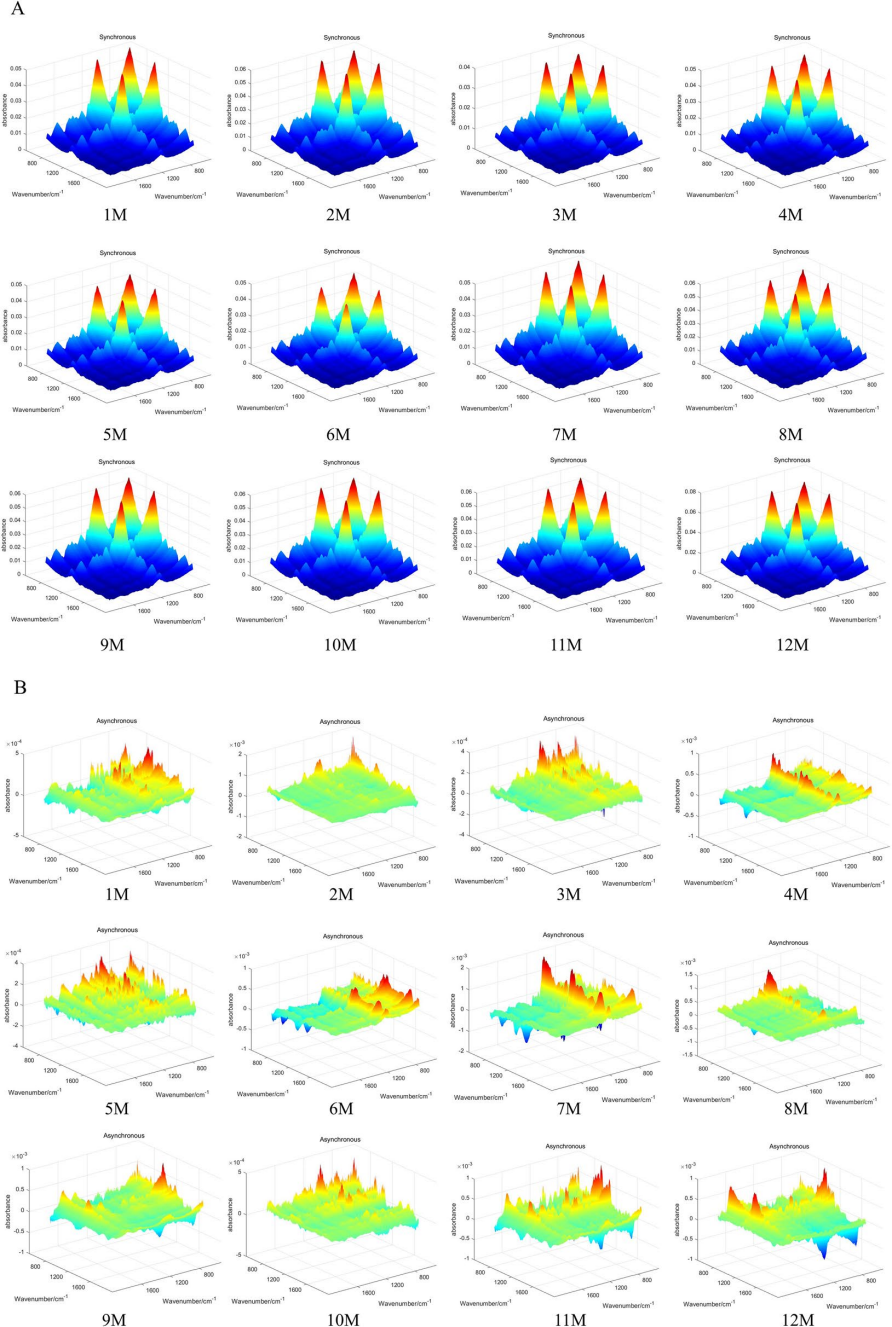

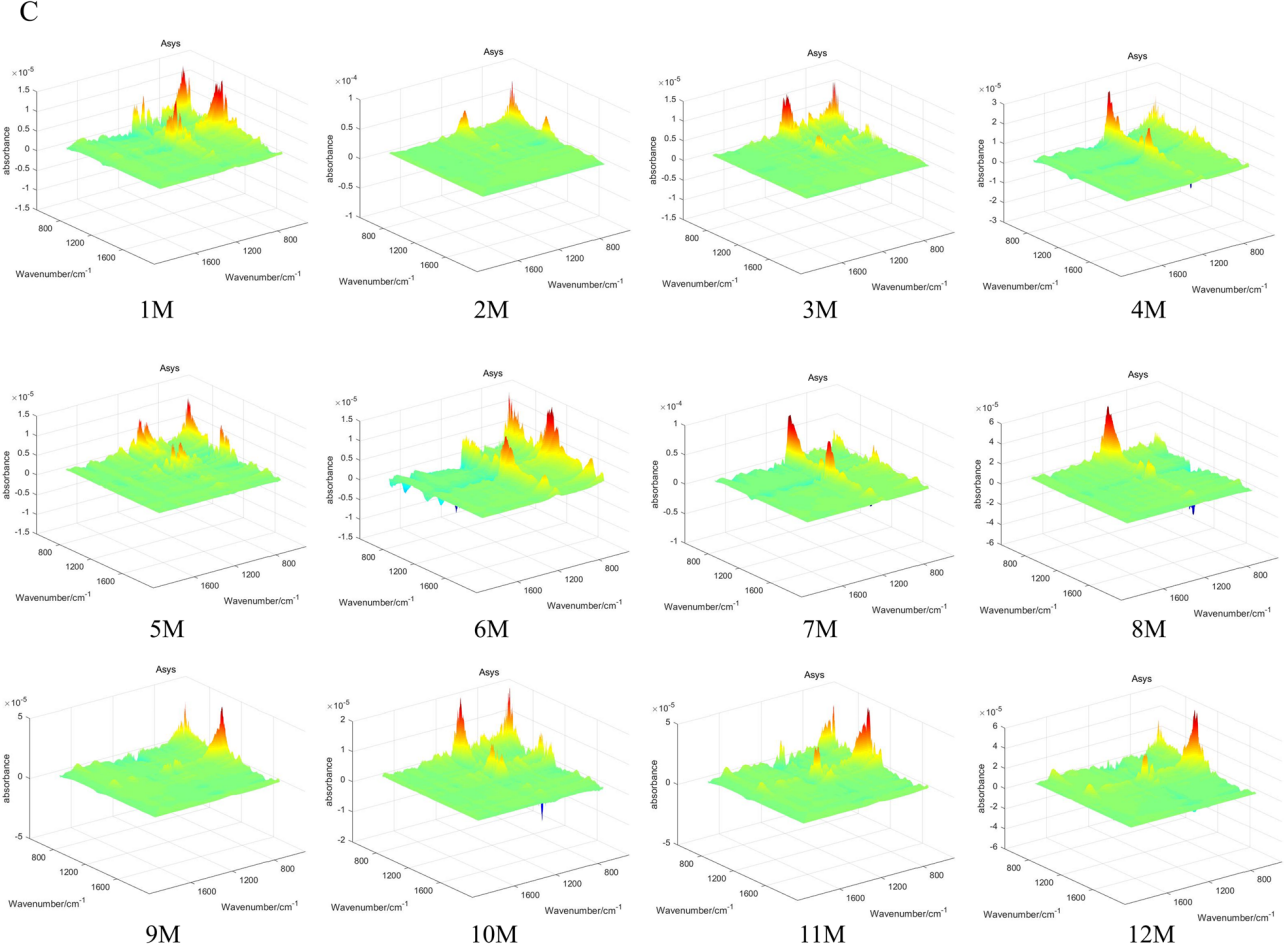
3DCOS of *D. officinale* stem. **A** synchronization; **B** synchronous; **C** synthesize

The 3DCOS plot has differences more obvious and clearer peak characteristics, mainly including position and intensity, while resolving spectral overlap and less obvious peak differences. In the synchronous 3DCOS of *D. officinale*, the absorbance of month 12 was significantly stronger than the other months; the asynchronous 3DCOS featured more peak information; and the integrated 3DCOS had the least spectral information (Fig. 4).

### Deep learning model results (ResNet)

**Fig. 5:**
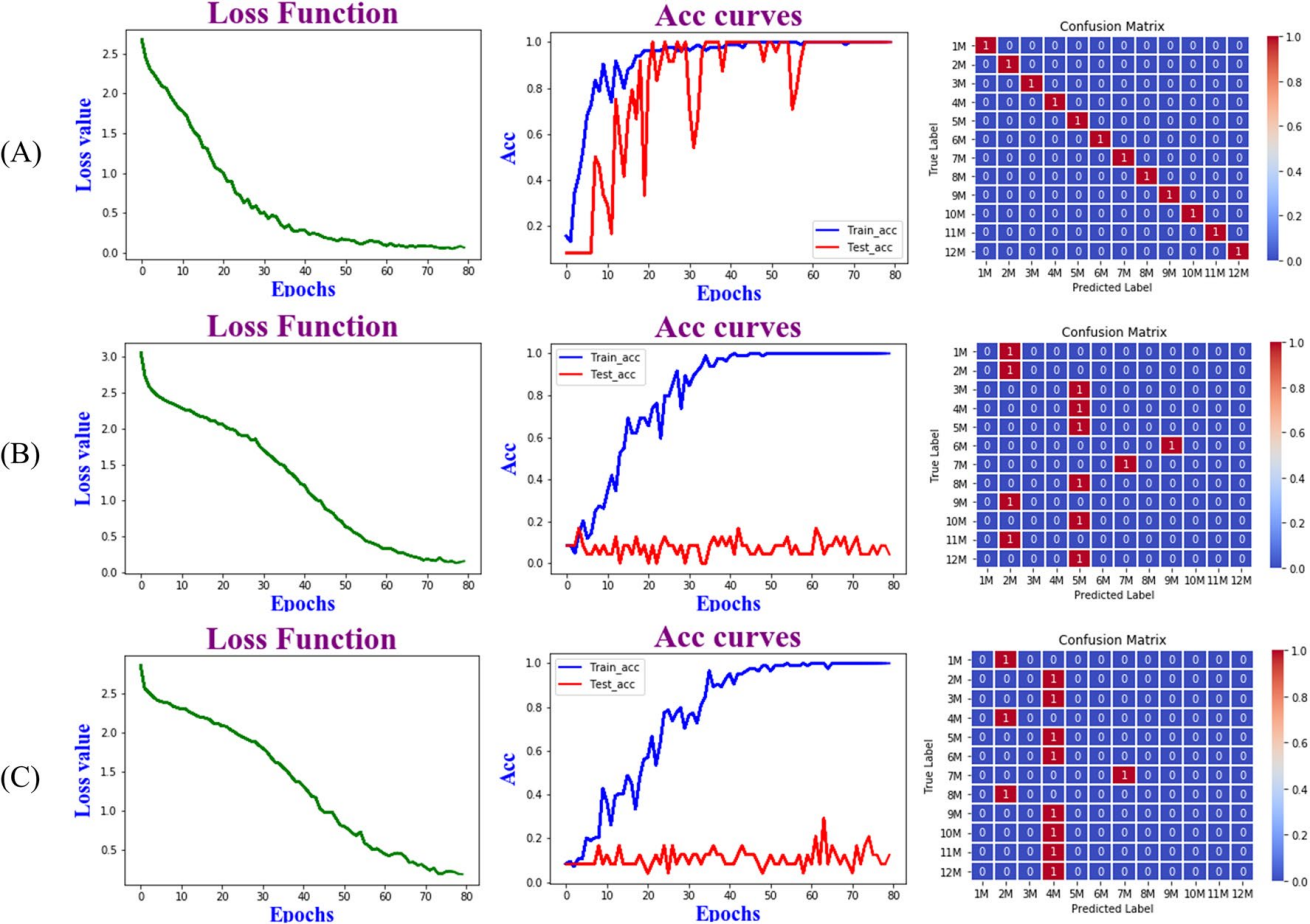
ResNet model based on 3DCOS. **A** synchronization; **B** synchronous; **C** synthesize

Based on the results of the above analysis, the ResNet model was further built with the weight decay coefficient γ were 0.0001 and the learning rate was 0.01. The model was constructed from synchronous, asynchronous and integrated 3DCOS image datasets of stems to identify *D. officinale* samples from different harvesting periods. The best synchronous 3DCOS results could be seen in Fig. 5A, with 100% accuracy in both training and test sets when the number of iterations was 58, with a loss value of 0.139, and 100% accuracy in external validation, with a total time of 9.8 min.

### Phenotypic data analysis

From the matrix of correlation coefficients and variance contribution of phenotypic traits, it could be seen that the three principal factors contributed the most to the explanatory variables with a cumulative contribution of 91.576%, which represented the information of *D. officinale* traits X1-X9 in the 12 months (Additional file 1: Table S2). In the rotated factor loading matrix, the 1st principal factor mainly contained the phenotypic trait information of X1, X2, X3, X4 and X5; the 2nd principal factor mainly contained the phenotypic trait information of X6 and X8; and the 3rd principal factor mainly contained the phenotypic trait information of X7 and X9 (Additional file 1: Table S3).The rankings of the composite factor scores in the 12 months of *D. officinale* were, in descending order: 11 > 3 > 12 > 1 > 4 > 5 > 9 > 6 > 2 > 8 > 10 > 7 (Table 3). It is worth noting that its harvesting period was from November to April of the following year, and all other months ranked within the top 5 except for the 9th ranked in the 2nd month, indicating that the factor analysis can be utilized to initially identify the suitable harvesting period of *D. officinale*.

**Table 3:** Composite factor score

Month	First principal factor	Second principal factor	Third principal factor	Aggregate score	NO
1	0.372897	0.241814	− 0.43721	0.134544	4
2	− 0.91393	0.418097	0.513253	− 0.2099	9
3	0.01221	0.768536	0.610066	0.358308	2
4	− 0.43336	1.01551	0.251237	0.113335	5
5	− 0.1055	− 0.30795	0.220011	− 0.07556	6
6	− 0.23098	− 0.35753	− 0.00096	− 0.20556	8
7	− 0.88661	− 0.05651	− 0.2763	− 0.51833	12
8	0.038571	− 0.76199	− 0.33093	− 0.26129	10
9	− 0.01105	− 0.35787	− 0.01912	− 0.10258	7
10	− 0.35179	− 0.40248	− 0.23974	− 0.33658	11
11	1.335152	− 0.46284	1.012049	0.789642	1
12	1.174386	0.263207	− 1.30236	0.313964	3

### Analysis of the coefficient of variation

Most of the *CV* of different months of *D. officinale* in traits X1-X5 were greater than 20%, implying that the data were unstable and varied greatly; in X6, the *CV* of March, July, August October and November were greater than 20%, indicating that the data of the samples in these months were unstable and varied greatly, with the greatest variability in July, with a *CV* value of 33%; in X7, only the data of March were unstable, with a *CV* value of 22%; and the information of X8 and X9 was the most stable, with the *CV* of less than 20% (Additional file 1: Table S4).

### Comparison of production in different months

**Fig. 6:**
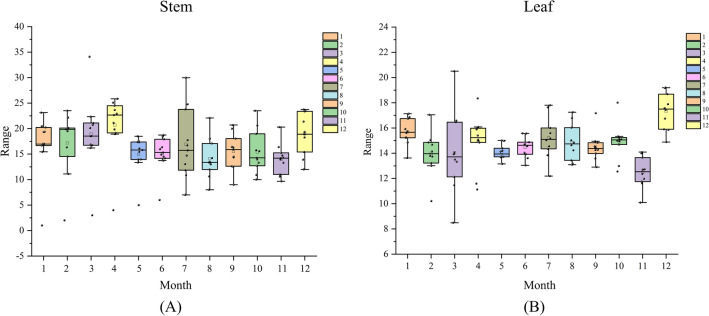
Dry matter content in different months of *D. officinale*. **A** Dry matter content of *dendrobium* stem; **B** Dry matter content of *dendrobium* leaf

Through the dry matter content can be used as an important indicator to judge the level of yield of *D. officinale* in different months, in general, its dry matter content was higher in November–April than other periods, with stems having the highest DMC in April and leaves having the highest in December, indicating that *D. officinale* had a higher yield in April and December (Fig. 6).

### Model parameters for PLSR

**Fig. 7:**
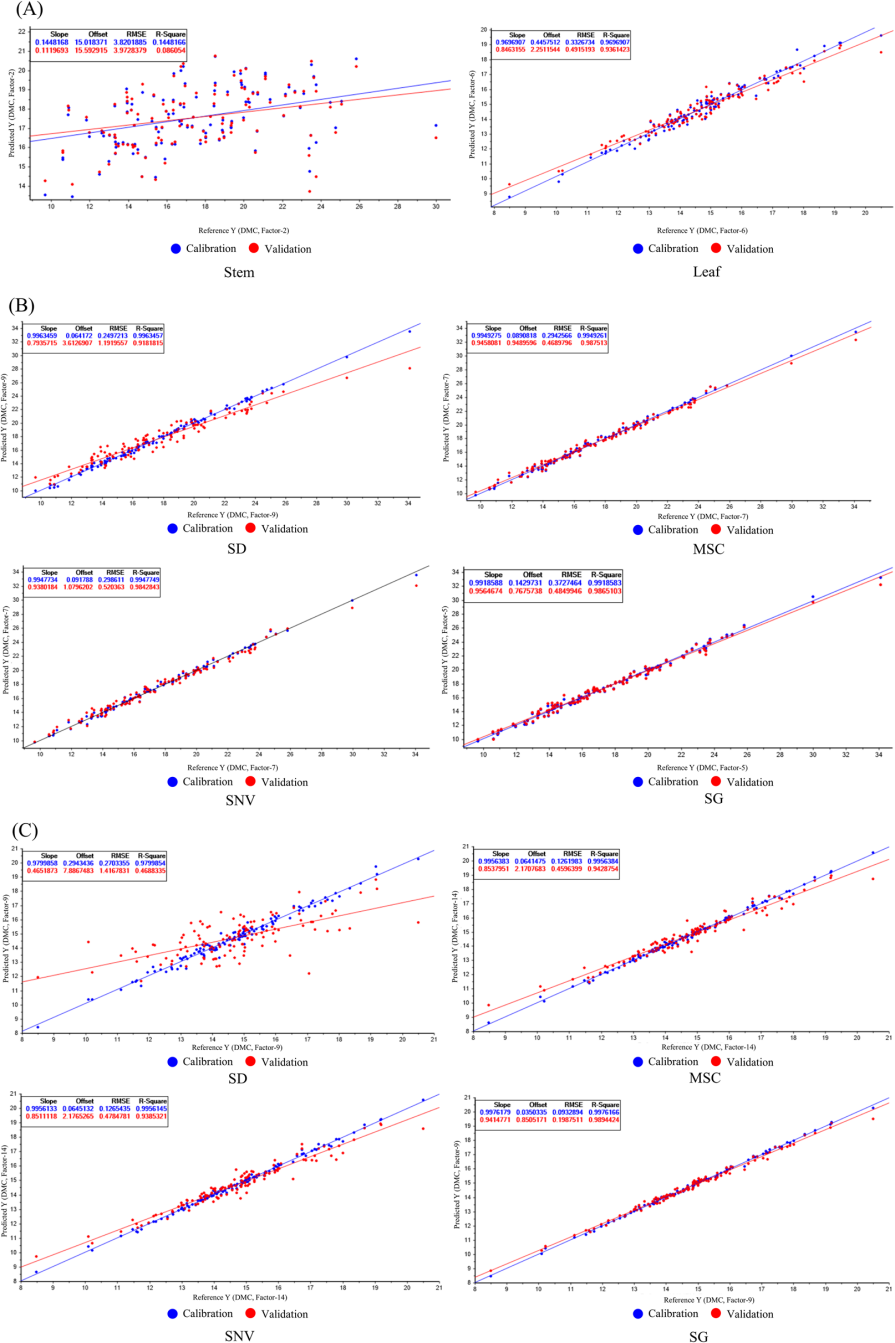
PLSR modeling of *Dendrobium officinale* after different pre-treatments. **A** Modeling with RAW; **B** PLSR modeling of *Dendrobium officinale* stems; **C** PLSR modeling of *Dendrobium officinale* Leaf

Predictive analysis of the dry matter content in different months of *D. officinale* can be used as a reliable method for evaluating its suitable harvesting period. Figure 7A represents the PLSR predictive model of dry matter content using raw data, with poor prediction of *D. officinale* stems and better fitting of the PLSR model for leaves. Considering the effects caused by different preprocessing on the model, the spectral data of *D. officinale* stem and leaf were further modeled after preprocessing (Fig. 7B, C). The parameters after modeling were shown in Table 4, the PLSR model built after preprocessing by MSC predicted the best dry matter content of *Dendrobium* stems with Factor = 7, Slope = 0.95, RMSE = 0.47, R^2^ = 0.99, and RPD = 8.79; and the PLSR model built after preprocessing by SG predicted the best dry matter content of *Dendrobium* leaves with Factor = 9, Slope = 0.94, RMSE = 0.2, R^2^ = 0.99, RPD = 9.55; it proves that the model established by this method has a stable effect, high precision, small error, and can predict the dry matter content of *D. officinale* stem and leaf at the same time.

**Table 4:** PLSR model parameters

Parts	Pretreatment methods	Factors	Slope	RMSE	R^2^	RPD
Stem	RAW	2	0.14	3.97	0.09	1.04
	SD	9	0.79	1.19	0.92	3.47
	**MSC**	**7**	**0.95**	**0.47**	**0.99**	**8.79**
	SNV	7	0.94	0.52	0.98	7.94
	SG	5	0.96	0.48	0.99	8.6
Leaf	RAW	6	0.85	0.94	0.49	2.03
	SD	9	0.47	1.42	0.47	1.35
	MSC	14	0.85	0.46	0.94	4.15
	SNV	14	0.85	0.49	0.94	3.9
	**SG**	**9**	**0.94**	**0.2**	**0.99**	**9.55**

Bolded values represent the models with the best result

### Effect of solar radiation (Sard) and precipitation (Pre) on the traits of *D. officinale* during november–april following year

Using Spearman's correlation analysis to correlate the *D. officinale* trait data from November to April with the corresponding solar radiation and precipitation, it was found that X2 was significantly negatively correlated with Sard, X6 was significantly positively correlated with Pre, and X8 was significantly negatively correlated with Pre within the appropriate harvesting period (Additional file 1: Fig. S3).

## Discussion

### Analysis of ATR-FTIR spectra of *D. officinale*

*Dendrobium* leaves were overall stronger than stems in terms of absorbance intensity in the characteristic peak 1750–1500 cm^−1^ range, which may be caused by the higher polysaccharide content of leaves than stems. The characteristic peak at 3417 cm^−1^ was the O–H telescopic vibrational absorption of polysaccharides; and the characteristic peaks in the range of 3000–2750 cm^−1^ were the methyl C–H anti-symmetric and symmetric telescopic vibrational absorption and the methylene-cyclohexane antisymmetric telescopic vibrational absorption. The characteristic peaks in the range 3000–2750 cm^−1^ are methyl C–H anti-symmetric and symmetric telescopic vibrational absorption and methylene C-H anti-symmetric telescopic vibrational absorptimethylene-cyclohexaneon [8]. 1702 cm^−1^ is the carbonyl C=O telescopic vibrational absorption of saccharides [5]. 1595 cm^−1^ is mainly due to the telescopic vibration of the carboxylate ions [26]. The 2 characteristic peaks with moderate absorption intensities near 1440 and 1380 cm^−1^ belong to the C–H stretching vibration, in-plane bending vibration, and –CH_3_ scissor bending vibration, respectively; 1322 cm^−1^ The characteristic peaks near the vicinity characterize the hydroxyl O–H bending vibration with the amide III band absorption; 1270–1245 cm^−1^ characterize the amide III band characteristic absorption of saccharides with the C–O–C stretching vibration [27]. 1027 cm^−1^ near the vicinity represents the characteristic absorption peaks of the pyran ring, which come from the asymmetric vibrational absorption of the C–O–C glycosidic bond of the pyran ring and C–O–H stretching vibration, respectively. It is noteworthy that the absorbance intensity of the characteristic peaks at 1702 cm^−1^ and 1595 cm^−1^ may be related to galacturonic acid [28]. The absorbance intensity of the characteristic peak at 1027 cm^−1^ may be related to the high or low content of galactomannan [29].

### Identification of different harvesting periods of *D. officinale*

PLS-DA has certain statistical validation defects when dealing with multiple covariance and inhomogeneous distribution, this shortcoming can be compensated by utilizing SVM which has a unique advantage in solving problems such as nonlinear and high-latitude data, and the results of PLS-DA can be validated [30]. The results proved that the accuracy of the SVM model was low, and the SVM model based on the GA algorithm took 50.85 min to model using the original data, and 117.26 min after preprocessing, and the more samples, the longer the time consumed. The ResNet model based on synchronous 3DCOS did not need to be preprocessed, and it took 9.8 min to build the model using the original data with 100% accuracy in both the training and test sets, and 100% accuracy in external validation. ResNet took less than 10% of the time of the SVM model, and achieveds a good classification effect regardless of the size of the samples and the number of categories [31]. ATR -FTIR combined with chemometrics for qualitative analysis, because it is not possible to assess the quality and yield of the high and low, when dealing with samples can be measured in its morphological characteristics data, using factor analysis in statistics can be used to provide a comprehensive assessment of the different months of harvesting *D. officinale*, to provide a reasonable time of harvesting. In this study, 3DCOS images of *Dendrobium* stems were successfully used to construct a ResNet model to recognize *Dendrobiums* with different harvesting periods, which largely saves time and cost compared to SVM models. Unfortunately, the number of external validation samples used in this study to verify the stability and generalization ability of the ResNet model is small, and there is some chance in the recognition results, and the model will be further validated by increasing the sample size in the future. However, the results of this study can still provide a reference for the identification of *Dendrobium* harvesting period, and also provide a theoretical basis for the quality evaluation of medicinal plants.

### Analysis of the best harvesting period of *D. officinale*

The results of the rotated factor loading matrix showed that the first principal factor was mainly determined by traits X1-X5, with an explanation rate of 44.83%, which could explain half of the information of the samples; the second principal factor and the third principal factor were determined by X6 and X8, and X7 and X9, respectively, which could explain 23.63% and 23.12% of the information of the samples, respectively. Most of the coefficients of variation of the first principal factor traits X1-X5 were within 20–50%, which might be caused by the small sample size and different selection criteria when collecting *D. officinale* individuals. Different people collect *Dendrobium* with different judgment criteria, part of some people choose stem length or stem thickness as a subjective factor, which can not represent the content of its effective chemical composition and dry matter, so it can not be used to evaluate the yield of medicinal plants by sex trans X1-X5. DMC is a direct factor affecting the high or low yield, and the coefficients of variation of X6 and X7 are relatively stable [16]. The content of polysaccharides in *D. officinale* leaves is higher than that of stems, and it has anti-tumor and antihypertensive effects on the human body [32]. Therefore, it is necessary to combine both stem and leaf components to assess the yield. In Fig. 6, the DMC of *D. officinale* stems and leaves was higher in November–April compared to other months, and such a result is consistent with the factor composite scores. The DMC of the stem reaches its highest level in April and December, and the DMC of the leaves in December is much higher than in other months, indicating that November to April of the following year is the suitable harvest period for *D. officinale*, and December is the optimal harvest period. Spearman's correlation analysis showed that during the suitable harvesting period (November–April of the following year) of *D. officinale*, an increase in Sard would lead to a decrease in trait X2, and an increase in Pre would lead to an increase in trait X6, i.e., the loss of stem water content, which affects photosynthesis and the accumulation of DMC by the plant through water supply, and the magnitude of its accumulation can be directly reflected in trait X1 [33]. Therefore, the suitable harvesting period of *D. officinale* needs a certain degree of light shading during the period of high solar radiation (14:00–17:00), and at the same time ensure sufficient water in the morning (7:00–10:00) to ensure the normal photosynthesis and DMC accumulation. In addition, different planting environments may lead to different optimal harvesting periods of *D. officinale*. Based on the importance of DMC on the appropriate harvesting period of *D. officinale*, ATR-FTIR-based DMC prediction analysis can reduce the time for sample processing and its phenotypic data analysis, and the optimal harvesting period of *D. officinale* can be evaluated quickly and efficiently.

## Conclusion

In this study, the 3DCOS combined with the ResNet model was used for the first time to determine the harvest period of *D. officinale*. Morphological and environmental factors were combined to evaluate the optimal harvest period of *D. officinale*, and PLSR prediction was used to analyze dry matter content. The results showed that the ResNet model was effective, with 100% accuracy in training, testing, and external validation. In addition, the model construction time was 90% faster than traditional models, greatly saving time and cost. The suitable harvesting period for *D. officinale* is from November to April of the following year, with the best harvesting period being the 12th month. During the harvesting period, plants need to be covered with a certain degree of light every day and maintained in sufficient water to ensure their photosynthesis and dry matter content. PLSR modeling of *D. officinale* stems and leaves based on MSC and SG preprocessing, respectively, was the best and can be used as an effective means to predict their dry matter content. In this study, ATR-FTIR spectroscopy, 3-dimensional correlation analysis, image recognition, and chemometrics analysis were used to construct a comprehensive analysis method for *Dendrobium* harvesting period identification and yield prediction, which has the advantages of fast, non-destructive and green. It provides a scientific method for the identification of suitable harvesting period and yield prediction of *Dendrobium*, which can guide local growers to choose the suitable harvesting time and reduce the economic losses caused by human factors. Meanwhile, the method has strong identification and generalization ability and can be popularized and applied to the research of identification and yield prediction of medicinal plants' origin, parts and suitable harvesting period.

### Supplementary Information

**Additional file 1: Fig. S1.** 200 permutation test for PLS-DA model with SD preprocessing. **Fig.** **S2.** SVM modeling based on GA algorithm. **Fig. S3.** Influence of environmental variables on trait characteristics of Dendrobium officinale at the appropriate harvesting period. **Table S1.** Information on the biomass of samples from actual sampling sites in Yunnan Province. **Table S2.** Eigenvalues, contribution rates and cumulative contribution rates of the main factors by factor analysis. **Table S3.** Factor loading coefficients after rotation. **Table S4.** Coefficient of variation of traits in different months of *D. officinale*.

## Data Availability

Data will be made available on request.

## Abbreviations

D. officinale: Dendrobium officinale
ATR-FTIR: Attenuated total reflection Fourier-transform infrared
3DCOS: Three-dimensional correlation spectroscopy
ResNet: Residual networks
PLS-DA: Partial least squares discriminant analysis
PLSR: Partial least squares regression
SVM: Support vector machine
SD: Second derivative
MSC: Multiplicative scatter correction
SNV: Standard normal variate
SG: Savitzky-Golay
DMC: Dry matter content
