# Supplementary material for: Optimization of the selection of suitable harvesting periods for medicinal plants: taking Dendrobium officinale as an example
Source: Plant Methods. 2024 Mar 16;20:43. doi: 10.1186/s13007-024-01172-9 (PMC10943765; doi:10.1186/s13007-024-01172-9)
Supplement: Supplementary file 1 — Additional file 1: Fig. S1. 200 permutation test for PLS-DA model with SD preprocessing. Fig. S2. SVM modeling based on GA algorithm. Fig. S3. Influence of environmental variables on trait characteristics of Dendrobium officinale at the appropriate harvesting period. Table S1. Information on the biomass of samples from actual sampling sites in Yunnan Province. Table S2. Eigenvalues, contribution rates and cumulative contribution rates of the main factors by factor analysis. Table S3. Factor loading coefficients after rotation. Table S4. Coefficient of variation of traits in different months of D. officinale. [file 13007_2024_1172_MOESM1_ESM.docx]

**Optimization of the selection of suitable harvesting periods for medicinal plants: the example of *Dendrobium officinale***

Peiyuan Li ^a b^, Tao shen ^c^, Li Li ^a, *^, Yuanzhong Wang ^b, *^

^a^ College of Biology and Environmental Sciences of Hunan province, Jishou University, Jishou, 416000, China

^b^ Medicinal Plants Research Institute, Yunnan Academy of Agricultural Sciences, Kunming, 650200, China

^c^ College of Chemistry, Biological and Environment, Yuxi Normal University, Yuxi, 653100, Yunnan, China

**Correspondence**

**Li Li**, College of Biological and Environmental Sciences of Hunan Province, Jishou University, Jishou, 416000, China. E-mail: lilyjsu@126.com

**Yuanzhong Wang**, Medicinal Plants Research Institute, Yunnan Academy of Agricultural Sciences, Kunming, 650200, China. E-mail: [boletus@126.com](mailto:boletus@126.com)

**Fig. S1:** 200 permutation test for PLS-DA model with SD preprocessing

**Fig. S2:** SVM modeling based on GA algorithm

**Fig. S3:** Influence of environmental variables on trait characteristics of Dendrobium officinale at the appropriate harvesting period

**Table S1**

Information on the biomass of samples from actual sampling sites in Yunnan Province

**Table S2**

Eigenvalues, contribution rates and cumulative contribution rates of the main factors by factor analysis

**Table S3**

Factor loading coefficients after rotation

**Table S4**

Coefficient of variation of traits in different months of *D. officinale*


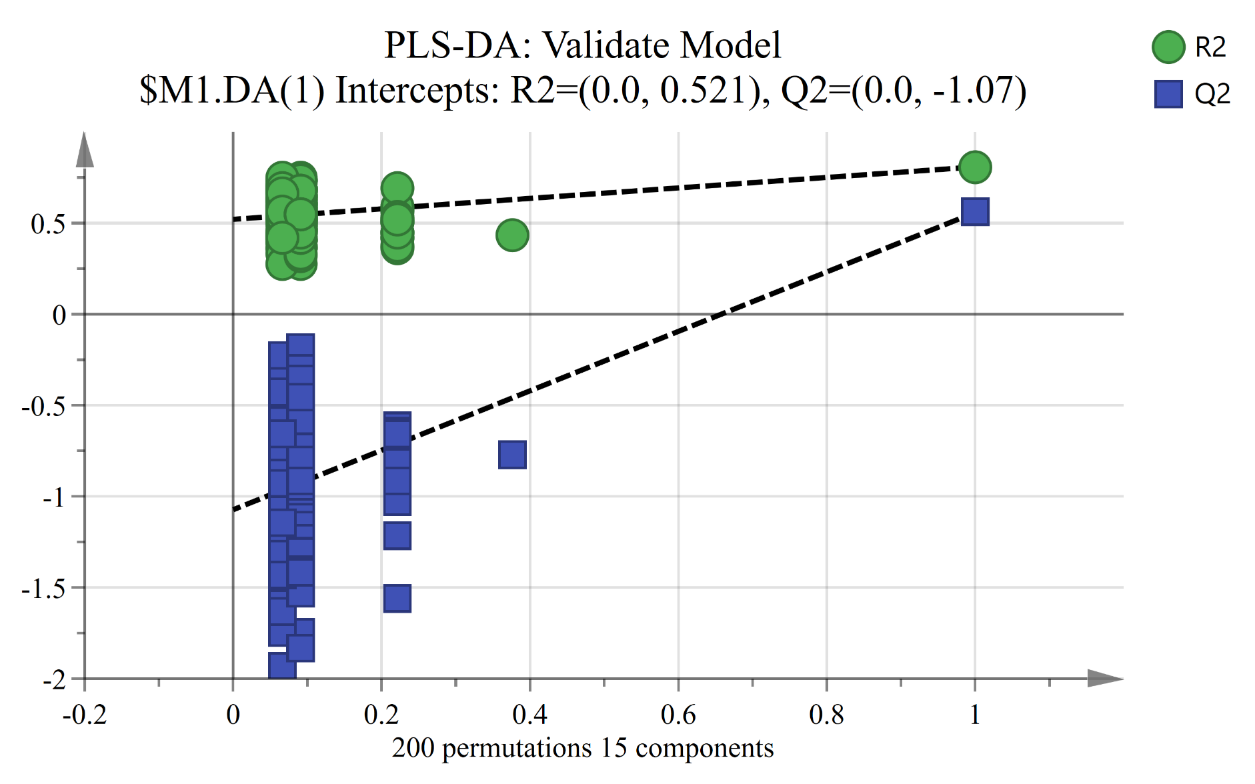


**Fig. S1** 200 permutation test for PLS-DA model with SD preprocessing


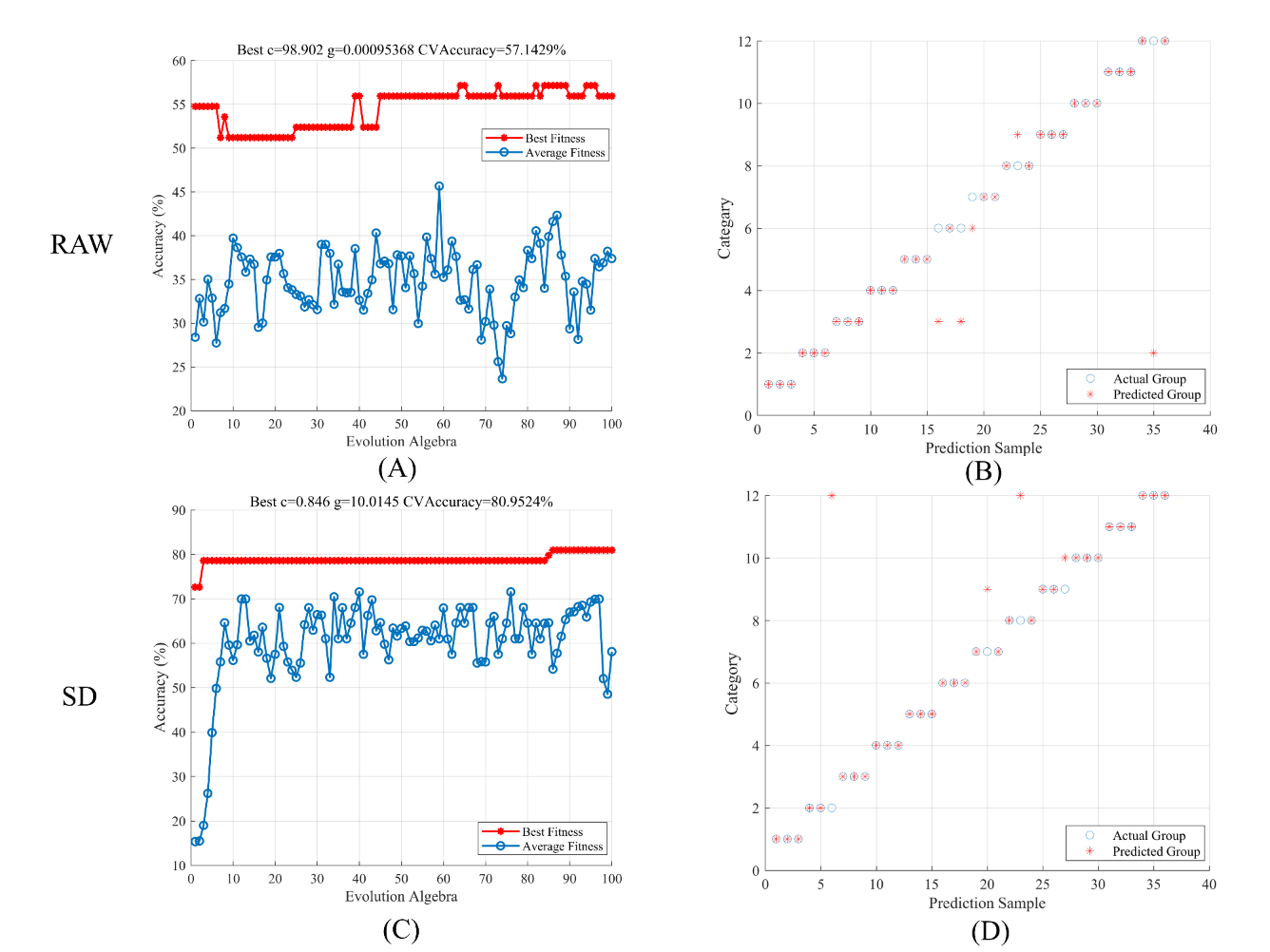


**Fig. S2** SVM modeling based on GA algorithm


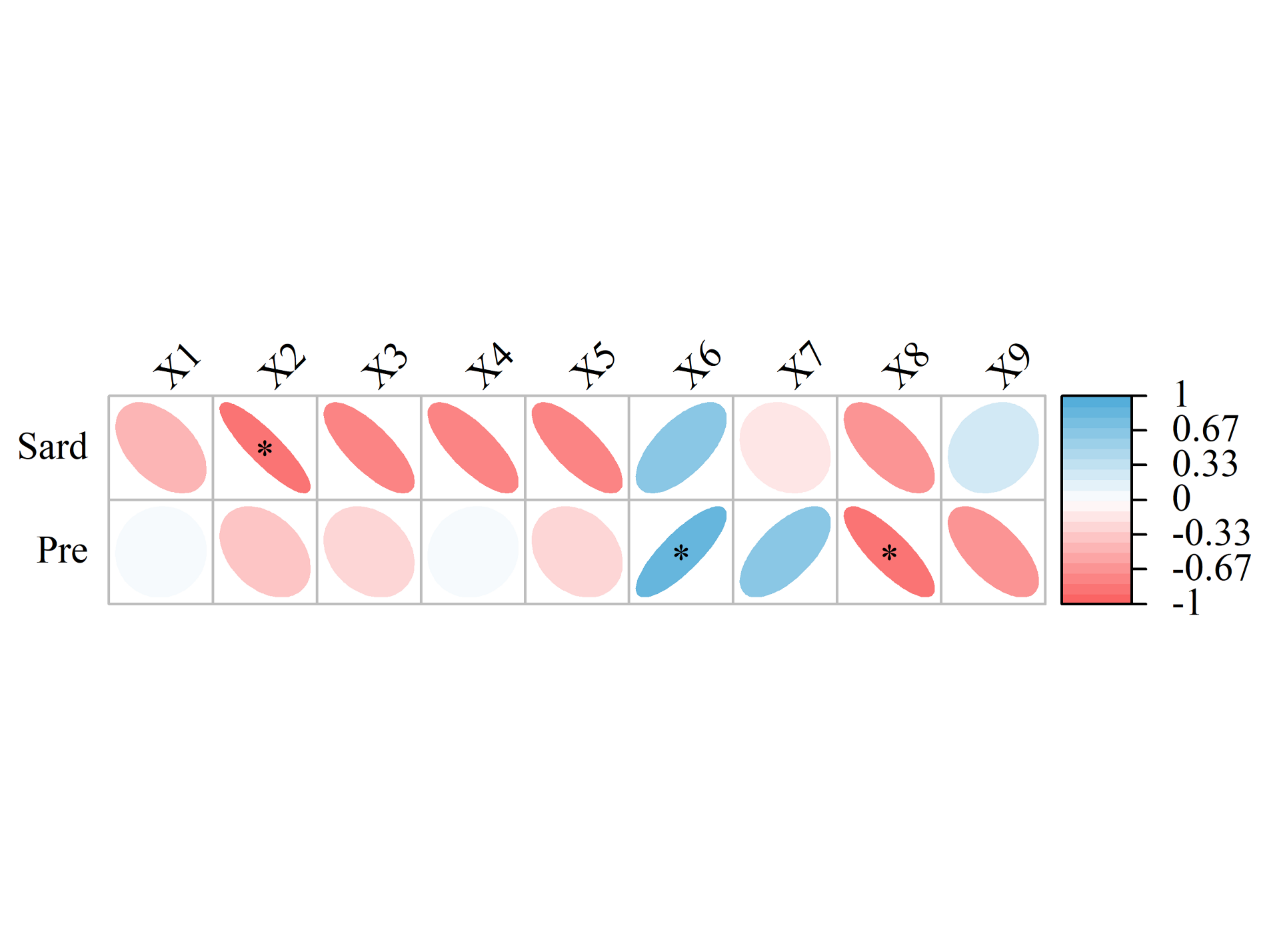


**Fig. S3** Influence of environmental variables on trait characteristics of *Dendrobium officinale* at the appropriate harvesting period

*: P<=0.05

**Table S1**

Information on the biomass of samples from actual sampling sites in Yunnan Province

| **Month** | **NO** | **X1** | **X2** | **X3** | **X4** | **X5** | **X6** | **X7** | **X8** | **X9** |
| --- | --- | --- | --- | --- | --- | --- | --- | --- | --- | --- |
| **1** | 1 | 16.00 | 5.74 | 3.12 | 0.97 | 0.47 | 16.89 | 15.22 | 83.11 | 84.78 |
|  | 2 | 21.50 | 6.86 | 2.95 | 1.06 | 0.40 | 15.45 | 13.63 | 84.55 | 86.37 |
|  | 3 | 27.90 | 8.47 | 3.79 | 1.71 | 0.59 | 20.23 | 15.61 | 79.77 | 84.39 |
|  | 4 | 19.30 | 4.64 | 3.34 | 1.07 | 0.56 | 23.13 | 16.86 | 76.87 | 83.14 |
|  | 5 | 16.10 | 6.94 | 4.72 | 1.18 | 0.75 | 16.96 | 15.92 | 83.04 | 84.08 |
|  | 6 | 15.70 | 6.29 | 3.76 | 1.06 | 0.63 | 16.78 | 16.74 | 83.22 | 83.26 |
|  | 7 | 14.40 | 3.77 | 2.66 | 0.77 | 0.42 | 20.48 | 15.72 | 79.52 | 84.28 |
|  | 8 | 17.30 | 5.56 | 4.37 | 1.08 | 0.67 | 19.34 | 15.26 | 80.66 | 84.74 |
|  | 9 | 16.70 | 5.75 | 4.10 | 1.11 | 0.61 | 19.38 | 14.86 | 80.62 | 85.14 |
|  | 10 | 17.70 | 4.59 | 2.67 | 0.77 | 0.46 | 16.68 | 17.13 | 83.32 | 82.87 |
| **2** | 1 | 8.20 | 2.49 | 1.65 | 0.55 | 0.28 | 22.16 | 17.05 | 77.84 | 82.95 |
|  | 2 | 14.80 | 4.30 | 2.40 | 0.87 | 0.36 | 20.11 | 14.86 | 79.89 | 85.14 |
|  | 3 | 10.80 | 2.48 | 1.50 | 0.49 | 0.21 | 19.86 | 14.13 | 80.14 | 85.87 |
|  | 4 | 11.00 | 3.27 | 1.94 | 0.77 | 0.27 | 23.51 | 13.74 | 76.49 | 86.26 |
|  | 5 | 11.80 | 3.51 | 1.86 | 0.51 | 0.26 | 14.52 | 13.80 | 85.48 | 86.20 |
|  | 6 | 12.00 | 3.00 | 2.67 | 0.59 | 0.35 | 19.51 | 13.22 | 80.49 | 86.78 |
|  | 7 | 9.70 | 3.48 | 2.70 | 0.39 | 0.28 | 11.11 | 10.21 | 88.89 | 89.79 |
|  | 8 | 9.70 | 2.53 | 1.38 | 0.50 | 0.18 | 19.90 | 13.01 | 80.10 | 86.99 |
|  | 9 | 10.40 | 3.32 | 1.96 | 0.54 | 0.29 | 16.33 | 14.68 | 83.67 | 85.32 |
|  | 10 | 9.50 | 2.45 | 0.96 | 0.49 | 0.14 | 20.04 | 15.05 | 79.96 | 84.95 |
| **3** | 1 | 27.40 | 3.86 | 4.31 | 0.82 | 0.52 | 21.11 | 12.13 | 78.89 | 87.90 |
|  | 2 | 41.10 | 8.74 | 6.65 | 1.47 | 0.90 | 16.87 | 13.50 | 83.13 | 86.50 |
|  | 3 | 19.50 | 3.50 | 2.35 | 1.19 | 0.39 | 34.10 | 16.46 | 65.90 | 83.50 |
|  | 4 | 20.80 | 4.80 | 3.21 | 0.89 | 0.43 | 18.53 | 13.33 | 81.47 | 86.70 |
|  | 5 | 22.40 | 3.22 | 4.24 | 0.65 | 0.49 | 20.11 | 11.48 | 79.89 | 88.50 |
|  | 6 | 30.70 | 4.24 | 3.85 | 0.88 | 0.33 | 20.74 | 8.49 | 79.26 | 91.50 |
|  | 7 | 21.20 | 3.51 | 2.64 | 0.57 | 0.54 | 16.20 | 20.50 | 83.80 | 79.50 |
|  | 8 | 13.30 | 3.73 | 2.02 | 0.83 | 0.28 | 22.34 | 13.93 | 77.66 | 86.10 |
|  | 9 | 19.20 | 3.04 | 2.46 | 0.56 | 0.41 | 18.53 | 16.58 | 81.47 | 83.40 |
|  | 10 | 19.30 | 2.50 | 2.35 | 0.42 | 0.33 | 16.74 | 14.04 | 83.26 | 86.00 |
| **4** | 1 | 38.90 | 5.79 | 3.71 | 1.15 | 0.68 | 19.84 | 18.35 | 80.16 | 81.65 |
|  | 2 | 32.10 | 3.57 | 3.45 | 0.90 | 0.52 | 25.09 | 14.99 | 74.91 | 85.01 |
|  | 3 | 22.10 | 2.83 | 3.44 | 0.64 | 0.51 | 22.66 | 14.85 | 77.34 | 85.15 |
|  | 4 | 22.60 | 2.94 | 3.03 | 0.69 | 0.48 | 23.65 | 15.89 | 76.35 | 84.11 |
|  | 5 | 21.50 | 2.79 | 2.69 | 0.59 | 0.41 | 21.12 | 15.40 | 78.88 | 84.60 |
|  | 6 | 14.10 | 3.37 | 4.17 | 0.64 | 0.48 | 18.92 | 11.59 | 81.08 | 88.41 |
|  | 7 | 19.60 | 2.71 | 3.07 | 0.52 | 0.34 | 19.16 | 11.12 | 80.84 | 88.88 |
|  | 8 | 17.50 | 2.23 | 1.17 | 0.55 | 0.18 | 24.50 | 15.07 | 75.50 | 84.93 |
|  | 9 | 15.20 | 1.71 | 0.82 | 0.44 | 0.13 | 25.84 | 15.98 | 74.16 | 84.02 |
|  | 10 | 14.60 | 1.36 | 1.10 | 0.31 | 0.18 | 22.92 | 16.09 | 77.08 | 83.91 |
| **5** | 1 | 21.50 | 4.33 | 4.22 | 0.67 | 0.55 | 15.46 | 13.16 | 84.54 | 86.84 |
|  | 2 | 32.50 | 5.50 | 4.27 | 1.02 | 0.59 | 18.49 | 13.75 | 81.51 | 86.25 |
|  | 3 | 23.30 | 4.02 | 3.74 | 0.70 | 0.56 | 17.37 | 14.98 | 82.63 | 85.02 |
|  | 4 | 24.10 | 4.17 | 3.25 | 0.73 | 0.45 | 17.55 | 13.72 | 82.45 | 86.28 |
|  | 5 | 23.60 | 3.03 | 2.79 | 0.49 | 0.38 | 16.11 | 13.73 | 83.89 | 86.27 |
|  | 6 | 20.90 | 2.77 | 2.83 | 0.37 | 0.41 | 13.34 | 14.39 | 86.66 | 85.61 |
|  | 7 | 30.70 | 4.92 | 4.30 | 0.78 | 0.59 | 15.81 | 13.69 | 84.19 | 86.31 |
|  | 8 | 23.50 | 3.77 | 3.57 | 0.53 | 0.51 | 13.94 | 14.14 | 86.06 | 85.86 |
|  | 9 | 24.50 | 2.58 | 1.90 | 0.45 | 0.28 | 17.31 | 15.01 | 82.69 | 84.99 |
|  | 10 | 19.90 | 2.51 | 2.45 | 0.35 | 0.35 | 13.96 | 14.20 | 86.04 | 85.80 |
| **6** | 1 | 29.80 | 4.65 | 5.09 | 0.64 | 0.72 | 13.72 | 14.03 | 86.28 | 85.97 |
|  | 2 | 30.50 | 4.64 | 3.82 | 0.76 | 0.57 | 16.35 | 14.88 | 83.65 | 85.12 |
|  | 3 | 22.00 | 2.90 | 2.73 | 0.44 | 0.40 | 15.32 | 14.70 | 84.68 | 85.30 |
|  | 4 | 29.70 | 4.50 | 3.24 | 0.64 | 0.42 | 14.17 | 13.05 | 85.83 | 86.95 |
|  | 5 | 31.30 | 4.82 | 3.89 | 0.77 | 0.57 | 15.97 | 14.57 | 84.03 | 85.43 |
|  | 6 | 19.80 | 3.14 | 1.94 | 0.59 | 0.30 | 18.73 | 15.58 | 81.27 | 84.42 |
|  | 7 | 24.50 | 2.69 | 2.08 | 0.50 | 0.32 | 18.45 | 15.56 | 81.55 | 84.44 |
|  | 8 | 23.80 | 2.92 | 2.82 | 0.43 | 0.41 | 14.90 | 14.72 | 85.10 | 85.28 |
|  | 9 | 24.40 | 2.92 | 2.45 | 0.52 | 0.34 | 17.91 | 13.92 | 82.09 | 86.08 |
|  | 10 | 18.10 | 1.70 | 1.83 | 0.25 | 0.25 | 14.51 | 13.61 | 85.49 | 86.39 |
| **7** | 1 | 38.20 | 2.51 | 2.74 | 0.62 | 0.41 | 24.77 | 15.03 | 75.23 | 84.97 |
|  | 2 | 18.70 | 2.30 | 2.36 | 0.27 | 0.29 | 11.84 | 12.19 | 88.16 | 87.81 |
|  | 3 | 20.30 | 1.32 | 1.50 | 0.23 | 0.22 | 17.75 | 14.55 | 82.25 | 85.45 |
|  | 4 | 20.80 | 3.16 | 2.76 | 0.34 | 0.38 | 10.88 | 13.85 | 89.12 | 86.15 |
|  | 5 | 17.60 | 1.60 | 0.68 | 0.21 | 0.11 | 13.04 | 15.60 | 86.96 | 84.40 |
|  | 6 | 23.30 | 1.69 | 1.73 | 0.51 | 0.31 | 29.98 | 17.80 | 70.02 | 82.20 |
|  | 7 | 14.90 | 1.15 | 1.20 | 0.17 | 0.17 | 14.72 | 14.35 | 85.28 | 85.65 |
|  | 8 | 22.10 | 1.95 | 2.15 | 0.46 | 0.34 | 23.77 | 16.00 | 76.23 | 84.00 |
|  | 9 | 21.80 | 1.89 | 2.21 | 0.30 | 0.39 | 15.73 | 17.64 | 84.27 | 82.36 |
|  | 10 | 20.60 | 2.27 | 1.70 | 0.38 | 0.26 | 16.72 | 15.17 | 83.28 | 84.83 |
| **8** | 1 | 36.40 | 2.28 | 2.15 | 0.30 | 0.28 | 13.35 | 13.09 | 86.65 | 86.91 |
|  | 2 | 34.80 | 2.85 | 2.53 | 0.38 | 0.38 | 13.37 | 15.02 | 86.63 | 84.98 |
|  | 3 | 31.40 | 2.96 | 2.39 | 0.53 | 0.40 | 18.02 | 16.79 | 81.98 | 83.21 |
|  | 4 | 31.40 | 2.91 | 2.53 | 0.64 | 0.44 | 22.09 | 17.25 | 77.91 | 82.75 |
|  | 5 | 29.00 | 3.18 | 3.49 | 0.45 | 0.51 | 14.22 | 14.64 | 85.78 | 85.36 |
|  | 6 | 23.00 | 2.56 | 2.21 | 0.35 | 0.33 | 13.49 | 14.85 | 86.51 | 85.15 |
|  | 7 | 25.60 | 2.76 | 2.67 | 0.47 | 0.38 | 17.08 | 14.23 | 82.92 | 85.77 |
|  | 8 | 38.10 | 5.45 | 5.82 | 0.65 | 0.78 | 11.98 | 13.44 | 88.02 | 86.56 |
|  | 9 | 45.50 | 6.48 | 7.21 | 0.69 | 0.95 | 10.62 | 13.18 | 89.38 | 86.82 |
|  | 10 | 26.80 | 3.81 | 3.74 | 0.50 | 0.60 | 13.00 | 16.03 | 87.00 | 83.97 |
| **9** | 1 | 33.20 | 3.46 | 3.55 | 0.72 | 0.50 | 20.71 | 13.98 | 79.29 | 86.02 |
|  | 2 | 44.30 | 4.51 | 3.26 | 0.72 | 0.46 | 15.90 | 14.25 | 84.10 | 85.75 |
|  | 3 | 27.10 | 3.95 | 2.47 | 0.71 | 0.35 | 18.03 | 14.28 | 81.97 | 85.72 |
|  | 4 | 32.40 | 5.53 | 4.72 | 0.91 | 0.64 | 16.42 | 13.58 | 83.58 | 86.42 |
|  | 5 | 23.30 | 2.78 | 2.72 | 0.45 | 0.40 | 16.29 | 14.83 | 83.71 | 85.17 |
|  | 6 | 29.40 | 3.93 | 3.26 | 0.50 | 0.47 | 12.62 | 14.46 | 87.38 | 85.54 |
|  | 7 | 30.60 | 3.96 | 3.28 | 0.57 | 0.49 | 14.36 | 14.95 | 85.64 | 85.05 |
|  | 8 | 27.70 | 3.20 | 4.65 | 0.46 | 0.60 | 14.50 | 12.90 | 85.50 | 87.10 |
|  | 9 | 21.60 | 1.86 | 1.87 | 0.37 | 0.32 | 19.98 | 17.18 | 80.02 | 82.82 |
|  | 10 | 28.20 | 3.97 | 3.21 | 0.50 | 0.47 | 12.51 | 14.54 | 87.49 | 85.46 |
| **10** | 1 | 25.30 | 4.98 | 3.49 | 1.03 | 0.51 | 20.61 | 14.69 | 79.39 | 85.31 |
|  | 2 | 32.30 | 4.68 | 4.26 | 0.89 | 0.64 | 18.98 | 15.11 | 81.02 | 84.89 |
|  | 3 | 31.20 | 3.76 | 4.45 | 0.41 | 0.56 | 10.91 | 12.55 | 89.09 | 87.45 |
|  | 4 | 22.30 | 2.60 | 2.63 | 0.41 | 0.40 | 15.75 | 15.25 | 84.25 | 84.75 |
|  | 5 | 22.10 | 2.91 | 2.21 | 0.41 | 0.33 | 13.95 | 15.00 | 86.05 | 85.00 |
|  | 6 | 20.10 | 2.63 | 1.68 | 0.33 | 0.25 | 12.71 | 14.96 | 87.29 | 85.04 |
|  | 7 | 26.30 | 2.33 | 2.03 | 0.36 | 0.31 | 15.56 | 15.11 | 84.44 | 84.89 |
|  | 8 | 17.30 | 3.41 | 2.43 | 0.80 | 0.44 | 23.49 | 18.01 | 76.51 | 81.99 |
|  | 9 | 16.40 | 3.65 | 2.67 | 0.52 | 0.35 | 14.27 | 12.99 | 85.73 | 87.01 |
|  | 10 | 16.90 | 2.43 | 1.79 | 0.32 | 0.28 | 13.30 | 15.33 | 86.70 | 84.67 |
| **11** | 1 | 42.20 | 8.43 | 8.17 | 1.24 | 1.04 | 14.74 | 12.71 | 85.26 | 87.29 |
|  | 2 | 32.00 | 7.22 | 6.84 | 0.96 | 0.80 | 13.30 | 11.75 | 86.70 | 88.25 |
|  | 3 | 42.20 | 7.40 | 6.74 | 1.50 | 0.95 | 20.30 | 14.09 | 79.70 | 85.91 |
|  | 4 | 20.70 | 5.31 | 3.86 | 0.76 | 0.54 | 14.32 | 13.99 | 85.68 | 86.01 |
|  | 5 | 32.40 | 7.69 | 6.21 | 1.08 | 0.85 | 13.99 | 13.66 | 86.01 | 86.34 |
|  | 6 | 28.30 | 9.03 | 4.90 | 0.96 | 0.61 | 10.59 | 12.37 | 89.41 | 87.63 |
|  | 7 | 28.90 | 5.21 | 5.23 | 0.85 | 0.67 | 16.38 | 12.73 | 83.62 | 87.27 |
|  | 8 | 28.40 | 5.86 | 6.61 | 0.89 | 0.67 | 15.25 | 10.09 | 84.75 | 89.91 |
|  | 9 | 48.50 | 8.20 | 9.55 | 1.16 | 1.11 | 14.20 | 11.62 | 85.80 | 88.38 |
|  | 10 | 23.30 | 5.43 | 3.50 | 0.53 | 0.42 | 9.67 | 11.97 | 90.33 | 88.03 |
| **12** | 1 | 24.90 | 7.95 | 3.98 | 1.11 | 0.67 | 13.91 | 16.74 | 86.09 | 83.26 |
|  | 2 | 37.30 | 6.38 | 4.82 | 1.52 | 0.90 | 23.75 | 18.68 | 76.25 | 81.32 |
|  | 3 | 26.90 | 5.56 | 2.87 | 1.08 | 0.45 | 19.34 | 15.85 | 80.66 | 84.15 |
|  | 4 | 30.80 | 4.75 | 3.75 | 0.90 | 0.66 | 18.91 | 17.57 | 81.09 | 82.43 |
|  | 5 | 40.20 | 8.57 | 7.53 | 2.00 | 1.35 | 23.38 | 17.89 | 76.62 | 82.11 |
|  | 6 | 25.00 | 4.84 | 2.76 | 0.91 | 0.53 | 18.89 | 19.20 | 81.11 | 80.80 |
|  | 7 | 22.40 | 3.56 | 2.83 | 0.83 | 0.54 | 23.43 | 19.17 | 76.57 | 80.83 |
|  | 8 | 13.90 | 5.86 | 3.27 | 1.07 | 0.52 | 18.27 | 15.90 | 81.73 | 84.10 |
|  | 9 | 17.80 | 4.04 | 2.27 | 0.62 | 0.34 | 15.42 | 14.89 | 84.58 | 85.11 |
|  | 10 | 80.30 | 16.19 | 7.61 | 3.46 | 1.33 | 21.37 | 17.43 | 78.63 | 82.57 |

Note: **X1**: Length of the stem (cm); **X2**: Fresh weight of stem (g); **X3**: Fresh weight of leaf (g); **X4**: Stem weights (g); **X5**: Leaf weights (g); **X6**: Dry matter content of stem (%); **X7**: Dry matter content of Leaf (%); **X8**: Water content of stem (%); **X9**: Water content of leaf (%).

**Table S2**

Eigenvalues, contribution rates and cumulative contribution rates of the main factors by factor analysis

| **Factor No.** | **Feature Root** | | | **Explanation of variance after rotation** | | |
| --- | --- | --- | --- | --- | --- | --- |
|  | **Feature Root** | **Explanation of variance %** | **Accumulation%** | **Feature Root** | **Explanation of variance %** | **Accumulation%** |
| 1 | 4.04 | 44.891 | 44.891 | 4.034 | 44.826 | 44.826 |
| 2 | 2.978 | 33.09 | 77.981 | 2.127 | 23.629 | 68.456 |
| 3 | 1.224 | 13.596 | 91.576 | 2.081 | 23.121 | 91.576 |

**Table S3**

Factor loading coefficients after rotation

| **Name** | **Factor loading factor** | | | **Common factor variance** |
| --- | --- | --- | --- | --- |
|  | **Factor 1** | **Factor 2** | **Factor 3** |  |
| X1 | **0.804** | -0.099 | -0.028 | 0.658 |
| X2 | **0.934** | -0.034 | 0.007 | 0.874 |
| X3 | **0.912** | -0.12 | 0.25 | 0.909 |
| X4 | **0.871** | 0.34 | -0.122 | 0.889 |
| X5 | **0.96** | -0.052 | -0.06 | 0.928 |
| X6 | -0.032 | **0.974** | -0.21 | 0.993 |
| X7 | -0.005 | 0.207 | **-0.978** | 0.999 |
| X8 | 0.032 | **-0.974** | 0.21 | 0.993 |
| X9 | 0.005 | -0.208 | **0.977** | 0.999 |

Note: **X1**: Length of the stem (cm); **X2**: Fresh weight of stem (g); **X3**: Fresh weight of leaf (g); **X4**: Stem weights (g); **X5**: Leaf weights (g); **X6**: Dry matter content of stem (%); **X7**: Dry matter content of Leaf (%); **X8**: Water content of stem (%); **X9**: Water content of leaf (%).

**Table S4**

Coefficient of variation of traits in different months of *D. officinale*

| **Month** | **Indicators** | **X1** | **X2** | **X3** | **X4** | **X5** | **X6** | **X7** | **X8** | **X9** |
| --- | --- | --- | --- | --- | --- | --- | --- | --- | --- | --- |
| **1** | Mean | 18.26 | 5.86 | 3.55 | 1.08 | 0.56 | 18.53 | 15.70 | 81.47 | 84.30 |
|  | SD | 3.73 | 1.29 | 0.68 | 0.25 | 0.11 | 2.24 | 1.00 | 2.24 | 1.00 |
|  | CV | 0.20 | 0.22 | 0.19 | 0.23 | 0.20 | 0.12 | 0.06 | 0.03 | 0.01 |
| **2** | Mean | 10.79 | 3.08 | 1.90 | 0.57 | 0.26 | 18.70 | 13.97 | 81.30 | 86.03 |
|  | SD | 1.72 | 0.58 | 0.54 | 0.13 | 0.06 | 3.51 | 1.66 | 3.51 | 1.66 |
|  | CV | 0.16 | 0.19 | 0.28 | 0.23 | 0.23 | 0.19 | 0.12 | 0.04 | 0.02 |
| **3** | Mean | 23.49 | 4.11 | 3.41 | 0.83 | 0.46 | 20.53 | 14.04 | 79.47 | 85.96 |
|  | SD | 7.39 | 1.66 | 1.34 | 0.30 | 0.17 | 4.92 | 3.10 | 4.92 | 3.10 |
|  | CV | 0.31 | 0.40 | 0.39 | 0.36 | 0.37 | 0.24 | 0.22 | 0.06 | 0.04 |
| **4** | Mean | 21.82 | 2.93 | 2.66 | 0.64 | 0.39 | 22.37 | 14.93 | 77.63 | 85.07 |
|  | SD | 7.60 | 1.15 | 1.14 | 0.22 | 0.17 | 2.37 | 2.02 | 2.37 | 2.02 |
|  | CV | 0.35 | 0.39 | 0.43 | 0.34 | 0.44 | 0.11 | 0.14 | 0.03 | 0.02 |
| **5** | Mean | 24.45 | 3.76 | 3.33 | 0.61 | 0.47 | 15.93 | 14.08 | 84.07 | 85.92 |
|  | SD | 3.86 | 0.97 | 0.79 | 0.20 | 0.10 | 1.67 | 0.56 | 1.67 | 0.56 |
|  | CV | 0.16 | 0.26 | 0.24 | 0.33 | 0.21 | 0.10 | 0.04 | 0.02 | 0.01 |
| **6** | Mean | 25.39 | 3.49 | 2.99 | 0.55 | 0.43 | 16.00 | 14.46 | 84.00 | 85.54 |
|  | SD | 4.46 | 1.02 | 0.98 | 0.15 | 0.14 | 1.72 | 0.77 | 1.72 | 0.77 |
|  | CV | 0.18 | 0.29 | 0.33 | 0.27 | 0.33 | 0.11 | 0.05 | 0.02 | 0.01 |
| **7** | Mean | 21.83 | 1.98 | 1.90 | 0.35 | 0.29 | 17.92 | 15.22 | 82.08 | 84.78 |
|  | SD | 5.92 | 0.57 | 0.63 | 0.14 | 0.09 | 5.94 | 1.60 | 5.94 | 1.60 |
|  | CV | 0.27 | 0.29 | 0.33 | 0.40 | 0.31 | 0.33 | 0.11 | 0.07 | 0.02 |
| **8** | Mean | 32.20 | 3.52 | 3.47 | 0.50 | 0.50 | 14.72 | 14.85 | 85.28 | 85.15 |
|  | SD | 6.36 | 1.30 | 1.63 | 0.13 | 0.20 | 3.22 | 1.39 | 3.22 | 1.39 |
|  | CV | 0.20 | 0.37 | 0.47 | 0.26 | 0.40 | 0.22 | 0.09 | 0.04 | 0.02 |
| **9** | Mean | 29.78 | 3.72 | 3.30 | 0.59 | 0.47 | 16.13 | 14.50 | 83.87 | 85.50 |
|  | SD | 5.94 | 0.94 | 0.84 | 0.16 | 0.09 | 2.66 | 1.06 | 2.66 | 1.06 |
|  | CV | 0.20 | 0.25 | 0.25 | 0.27 | 0.19 | 0.16 | 0.07 | 0.03 | 0.01 |
| **10** | Mean | 23.02 | 3.34 | 2.76 | 0.55 | 0.41 | 15.95 | 14.90 | 84.05 | 85.10 |
|  | SD | 5.41 | 0.89 | 0.93 | 0.24 | 0.12 | 3.71 | 1.39 | 3.71 | 1.39 |
|  | CV | 0.24 | 0.27 | 0.34 | 0.44 | 0.29 | 0.23 | 0.09 | 0.04 | 0.02 |
| **11** | Mean | 32.69 | 6.98 | 6.16 | 0.99 | 0.76 | 14.27 | 12.50 | 85.73 | 87.50 |
|  | SD | 8.46 | 1.34 | 1.77 | 0.26 | 0.21 | 2.79 | 1.17 | 2.79 | 1.17 |
|  | CV | 0.26 | 0.19 | 0.29 | 0.26 | 0.28 | 0.20 | 0.09 | 0.03 | 0.01 |
| **12** | Mean | 31.95 | 6.77 | 4.17 | 1.35 | 0.73 | 19.67 | 17.33 | 80.33 | 82.67 |
|  | SD | 17.82 | 3.48 | 1.83 | 0.79 | 0.34 | 3.19 | 1.40 | 3.19 | 1.40 |
|  | CV | 0.56 | 0.51 | 0.44 | 0.59 | 0.47 | 0.16 | 0.08 | 0.04 | 0.02 |
